# ESO-Based Non-Singular Terminal Filtered Integral Sliding Mode Backstepping Control for Unmanned Surface Vessels

**DOI:** 10.3390/s25020351

**Published:** 2025-01-09

**Authors:** Jianping Yuan, Zhuohui Chai, Qingdong Chen, Zhihui Dong, Lei Wan

**Affiliations:** 1 Naval Architecture and Shipping College, Guangdong Ocean University, Zhanjiang 524091, China; 2112212008@stu.gdou.edu.cn (Z.C.); 2112212003@stu.gdou.edu.cn (Q.C.); dongzhihui@gdou.edu.cn (Z.D.); 2 College of Shipbuilding Engineering, Harbin Engineering University, Harbin 150001, China; wanlei@hrbeu.edu.cn; 3 Guangdong Provincial Key Laboratory of Intelligent Equipment for South China Sea Marine Ranching, Guangdong Ocean University, Zhanjiang 524088, China; 4 Guangdong Provincial Engineering Research Center for Ship Intelligence and Safety, Zhanjiang 524000, China

**Keywords:** unmanned surface vessels, ESO, heading tracking, path tracking, non-singular terminal sliding mode

## Abstract

Aiming at the control challenges faced by unmanned surface vessels (USVs) in complex environments, such as nonlinearities, parameter uncertainties, and environmental perturbations, we propose a non-singular terminal integral sliding mode control strategy based on an extended state observer (ESO). The strategy first employs a third-order linear extended state observer to estimate the total disturbances of the USV system, encompassing both external disturbances and internal nonlinearities. Subsequently, a backstepping sliding mode controller based on the Lyapunov theory is designed to generate the steering torque control commands for the USV. To further enhance the tracking performance of the system, we introduce a non-singular terminal integral sliding mode surface with a double power convergence law and redesign the backstepping sliding mode controller for the USV heading control. Meanwhile, to circumvent the differential explosion issue in traditional backstepping control, we simplify the controller design by utilizing a second-order sliding mode filter to accurately estimate the differential signals of the virtual control quantities. Theoretical analysis and simulation results demonstrate that the proposed control algorithm improves the convergence speed, adaptive ability, and anti-interference ability in complex environments compared to traditional linear backstepping sliding mode control, thereby enhancing its engineering practicability. This research offers a more efficient and reliable method for precise heading control and path tracking of USVs in complex and dynamic environments.

## 1. Introduction

In recent years, unmanned systems technology has been subjected to unprecedented in-depth research efforts. In this context, unmanned surface vessels (USVs) have been widely utilized in both military and civilian projects, owing to their unique advantage of being able to operate in harsh environments [1]. In the military field, USVs have been applied to tasks such as alert patrol, anti-submarine warfare, and anti-mine warfare, among others. In the civilian sector, the USV, with its compact displacement and unique shallow-draft design, has found widespread use in sea ranch channel mapping, underwater geomorphology mapping, water quality monitoring, and sampling, among other tasks [2].

Due to marine environment complexity, USVs are prone to external interference, which risks failure or danger [3]. Thus, accurate and stable USV control systems are crucial [4]. Current designs rely on model control, but obtaining certain hydrodynamic parameters is challenging, especially with limited experiments [5]. Hence, addressing model inaccuracies and external disturbances is a key research focus.

Addressing the challenges posed by external environmental disturbances and inaccuracies in USV control systems, methods to observe or approximate these disturbances and inaccuracies, and to compensate for them in the controller, have been intensively studied. Reference [6] successfully compensated for uncertainties by integrating backstepping with RBFNN, enabling precise trajectory tracking. Reference [7] utilized a self-organizing neural network to approximate complex nonlinear functions, mitigating the negative effects of model uncertainty, environmental perturbations, and actuator failures. In [8], an enhanced NDO-based MPC method was introduced to address USV trajectory tracking issues under wind and wave currents, demonstrating superior performance and reduced parameter tuning needs. Reference [9] presented a tracking error state based on integral line-of-sight guidance and designed a variable gain observer to ensure stability and tracking accuracy. The authors in [10] proposed an optimal control scheme using backstepping and a disturbance observer for ship stability, comparing it favorably with traditional controllers. Reference [11] employed a reduced order ESO to solve the time-varying sideslip angle problem under external disturbances. Reference [12] developed an adaptive disturbance compensation control mechanism, verified through simulations and real-world experiments. In [13], an estimator for unknown system dynamics was designed to reconstruct uncertainty, aiding in USV trajectory tracking. Finally, ref. [14] introduced a novel disturbance observer to estimate and compensate for modeling uncertainties and external disturbances, showcasing fast response, improved transient performance, and robustness in simulations.

Meanwhile, sliding mode control, insensitive to external disturbances, is widely used in USV research. Reference [15] proposes a backstepping adaptive sliding mode controller, validated through theory and simulations. Reference [16] designs a discrete-time full-order sliding mode heading controller for target path tracking under interference. In [17], a Fixed Time Fractional Order Smooth Mode Control (FTFOSMC) strategy accelerates USV system convergence and ensures path tracking. Reference [18] uses neural networks, auxiliary dynamics, and backstepping for adaptive sliding mode control under modeling uncertainty, verified by simulations. Reference [19] designs a fixed-time sliding mode controller for USV trajectory tracking, showing strong robustness against uncertainties. Reference [20] controls the convergence time of the adaptive sliding mode controller via the Lyapunov function, and experiments on a USV prototype to confirm its effectiveness in stabilizing within the desired time.

Based on the above considerations, this paper proposes a non-singular terminal integral sliding-mode backstepping control scheme based on an extended state observer, which combines the extended state observer with a backstepping sliding-mode controller to generate a steering torque for tracking the desired heading and path; and to design novel sliding-mode surfaces and convergence laws, in addition to using second-order sliding-mode filters for estimating the differentials of the virtual control quantities, in order to reduce the complexity of the controller. The contributions of this paper are as follows:

(1) In order to address the shortcomings of the traditional sliding mode surface in terms of asymptotic tracking performance and stability during the control process, this paper redesigns and constructs a novel non-singular terminal integral sliding mode surface, which combines the advantages of the finite-time convergence property of the terminal sliding mode and the non-singularity, aiming to enhance the tracking performance and convergence rate of the system, and also effectively avoids the singularity problem that may occur in the traditional terminal sliding mode.
(2) Design ESO to estimate and output the aggregate disturbance in the system in real time. Through this design, we are able to take the complex system dynamics and uncertainty factors into account, and then improve the robustness and adaptability of the system through compensation strategies to enhance the stable operation of the system under various disturbance conditions.
(3) A second-order sliding mode filter (SOSMF) is introduced to estimate the differential signals of the virtual control volume. This design not only reduces the computational complexity, but also effectively avoids the problem of differential explosion due to continuous differential operation in the backstepping design, which makes it easier to ensure the application in real engineering.

## 2. Problem Formulation and Preliminaries

### 2.1. Mathematical Modeling of USV

**Figure 1:**
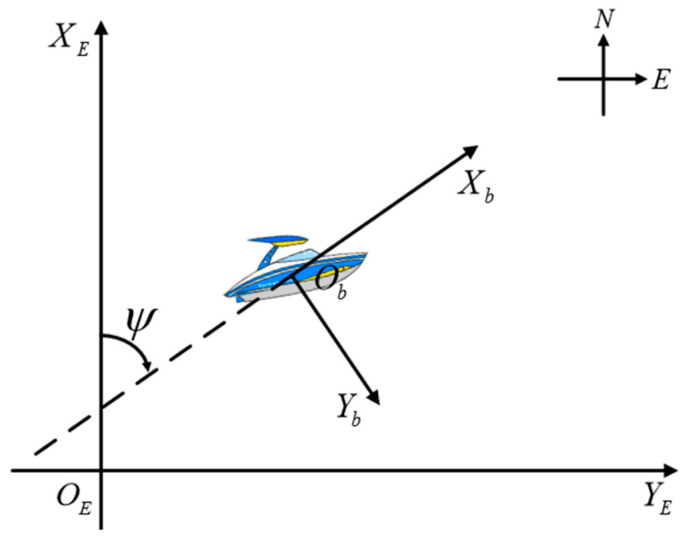
Schematic of USV model motion coordinate system.

In the realm of ship maneuvering research, the primary manifestations of ship motion within the horizontal plane encompass sway, roll, and yaw, which directly influence the vessel’s heading and position—factors of paramount importance to navigation safety. While trim, roll, and heave also exert an influence on ship performance, their impacts on navigation safety are comparatively minor and are generally considered secondary factors. Consequently, the conventional approach focuses predominantly on transverse sway, longitudinal sway, and yaw motions, while longitudinal oscillation, transverse oscillation, and pitch motions are typically overlooked. For USV, its motion situation also follows this simplification principle, which can be abstracted as a planar motion model containing only three degrees of freedom, with the introduction of a fixed coordinate system $\left\{X_{E}O_{E}Y_{E}\right\}$ and a follower coordinate system $\left\{X_{b}O_{b}Y_{b}\right\}$, as shown in Figure 1, and the following kinematic model is constructed on the basis of the above simplification idea:

(1) $$\left\{\begin{array}{l} \dot{x}=u\cos\psi -v\sin\psi \\ \dot{y}=u\sin\psi +v\cos\psi \\ \dot{\psi }=r \end{array}\right.$$

The USV dynamics model can be expressed as:

(2) $$\left\{\begin{array}{l} \dot{u}=\frac{m_{2}}{m_{1}}vr-\frac{d_{1}}{m_{1}}u+\frac{1}{m_{1}}\tau_{u}\\ \dot{v}=-\frac{m_{1}}{m_{2}}ur-\frac{d_{2}}{m_{2}}v\\ \dot{r}=\frac{m_{1}-m_{3}}{m_{3}}uv-\frac{d_{3}}{m_{3}}r+\frac{1}{m_{3}}\tau_{r}+\omega (t)+\Delta_{r} \end{array}\right.$$

The simplified USV horizontal plane motion coordinate system is shown below:

Where x and y denote the longitudinal and transverse positions of the USV in the geodetic coordinate system, ψ is the heading angle, u, v, and r denote the surge, sway, and yaw velocities, respectively. $m_{1}$, $m_{2}$, and $m_{3}$ are the model parameters of USV containing the added masses and moment of inertia, and the hydrodynamic damping are denoted by $d_{1}$, $d_{2}$, and $d_{3}$. $\tau_{u}$ represents the thrust generated by the propeller of the USV, and $\tau_{r}$ is the steering torque of the USV, respectively. ω(t) indicates an unknown external perturbation, $\Delta_{r}$ is the unmodeled dynamics in the system.

### 2.2. Preliminaries

**Lemma** **1.** *For the nonlinear system* $\dot{x}=f(x,t)$*, assume that there exists a first-order continuously differentiable positive definite function* V(x) *(satisfying* V(0)=0*), and that all nonzero points in the state space satisfy the following conditions:* V(x)>0 *and* $\dot{V}(x)<0$*,* $\left\|x\right\|\to 0$*,* V(x)→0*. then, the equilibrium state of this nonlinear system is asymptotically stable.*

**Lemma** **2 ([21]).** *For the nonlinear system* $\dot{x}=f(x,t)$*, if the system is stabilized within a bounded function* $T(x_{0})$ *related to the initial state of the system, i.e., there exists a time constant* $T_{\max}(x_{0})\in R^{+}$ *dependent on the initial state of the system such that* x=0 *for* t>T *and* $T<T_{\max}(x_{0})$*, then the system is said to be capable of converging to stability in a finite time.*

**Lemma** **3 ([22]).** *For the nonlinear system* $\dot{x}=f(x,t)$*, assume that there exists a continuous positive definite Lyapunov function* V(x) *with* V(0)=0 *and* V(x) *in a neighborhood* $O\subseteq A\subseteq R^{n}$ *of the origin satisfies:* $V(x)+\zeta V^{\kappa }(x)\leq 0$*, where* O→R*,* $\zeta \in R^{+}$ *and* κ∈(0,1)*. At this point, A finite time stabilized convergence time T is computed as:*

(3) $$T(x_{0})\leq \frac{1}{\zeta (1-\kappa )}V^{1-\kappa }(x(t_{0}))$$

**Hypothesis** **1.** *The actual USV can obtain its real-time status information, such as position and angular velocity, from the navigation sensors.*

**Hypothesis** **2.** *The external time-varying interference term* ω(t) *is bounded, but its upper bound is unknown, i.e.,* $\left|\omega (t)\right|\leq \omega {\left(t\right)}_{\max}<\infty$*.*

**Hypothesis** **3.** *The actual control loss term* $\tau_{r}$ *is bounded, but its upper bound is unknown, i.e.,* $\left|\tau_{r}\right|\leq \tau_{r,\max}<\infty$*.*

## 3. Control System Design

**Figure 2:**
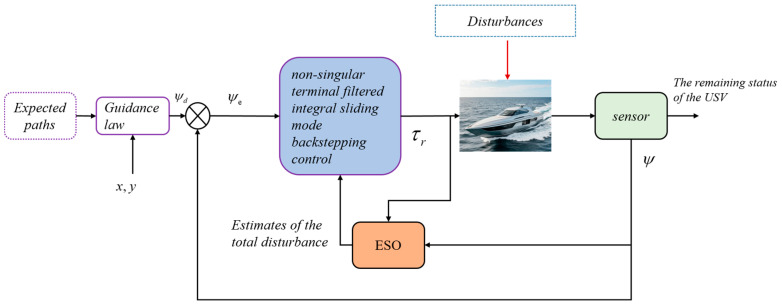
Controller structure diagram of this paper.

In this section, a control strategy is developed for USVs in complex environments, where first, the ESO is utilized to estimate the aggregate disturbances, which include the external disturbances as well as the model uncertainty term. Then, the estimated disturbances are fed back to the non-singular terminal sliding mode control system to keep it stable for a finite period of time. The specific design will be presented in subsequent Section 3.1 and Section 3.2. The control structure diagram is shown in Figure 2.

**Remark** **1.** *In the path tracking system, we use the basic guidance law in reference [23] to realize path tracking by converting the position error to the desired course angle* $\psi_{d}=\pi_{k}+\tan^{-1}(y_{e}/\Delta )$ *using the lateral displacement deviation, where* $\pi_{k}$ *is the azimuth,* $y_{e}$ *is the lateral deviation, and* Δ *is the forward looking distance.*

### 3.1. Design of ESO

The ESO can accurately track the motion state of the USV and estimate the approximate aggregate disturbance based on the control inputs and sensor outputs. For system (1) and (2), let $x_{1}=\psi$, $x_{2}=r$, then the input to the ESO is the steering torque of the USV, and the output is the heading angle acquired by the USV’s on-board sensors.

Based on the above description, the USV heading control system in the horizontal plane can be represented as the following second-order nonlinear system in the presence of disturbance and uncertainty terms:

(4) $$\left\{\begin{array}{l} {\dot{x}}_{1}=x_{2}\\ {\dot{x}}_{2}=D(x_{1},x_{2},\omega (t),\Delta_{r})+bu\\ y=x_{1}\\ \tau_{r}=u \end{array}\right.$$

For system (1), The following observer can be designed:

(5) $$\left\{\begin{array}{l} \tilde{\varepsilon }=x_{1}-{\hat{x}}_{1}\\ {\dot{\hat{x}}}_{1}={\hat{x}}_{2}-\varpi_{1}\tilde{\varepsilon }\\ {\dot{\hat{x}}}_{2}={\hat{x}}_{3}-\varpi_{2}\tilde{\varepsilon }+bu\\ {\dot{\hat{x}}}_{3}=-\varpi_{3}\tilde{\varepsilon } \end{array}\right.$$

where $\tilde{\varepsilon }$ is the error, ${\hat{x}}_{1}$ and ${\hat{x}}_{2}$ are estimates of $x_{1}$ and $x_{2}$, respectively, and ${\hat{x}}_{3}$ is the estimate of $D(x_{1},x_{2},\omega (t),\Delta_{r})$. From the literature [24], it can be seen that by designing a suitable observer gain $\varpi_{1}$, $\varpi_{2}$ and $\varpi_{3}$, these tracking errors would be small enough.

### 3.2. Design of the Controller

The controller in this paper is mainly divided into two steps, as follows:

**Step1.** Assuming that the commanded heading is $x_{d}$ and the actual heading is $x_{1}$, define the first error variable:

(6) $$e_{1}=x_{1}-x_{d}$$

Combining System (4) and taking the derivative of Equation (6), the derivative of the first error can be expressed as:

(7) $$\begin{array}{ll} {\dot{e}}_{1} & ={\dot{x}}_{1}-{\dot{x}}_{d}\\ & =x_{2}-{\dot{x}}_{d} \end{array}$$

At this point, construct the first Lyapunov function:

(8) $$V_{1}=\frac{e_{1}^{2}}{2}$$

Then, it is available:

(9) $$\begin{array}{l} {\dot{V}}_{1}=e_{1}{\dot{e}}_{1}\\ =e_{1}(x_{2}-{\dot{x}}_{d}) \end{array}$$

In order to ${\dot{V}}_{1}\leq 0$, define the intermediate dummy control variable ρ as:

(10) $$\rho =-\varphi_{1}e_{1}+{\dot{x}}_{d}$$

In Equation (10), $\varphi_{1}>0$. Taking the derivative with respect to the intermediate dummy control variable yields:

(11) $$\dot{\rho }=-\varphi_{1}e_{1}+{\ddot{x}}_{d}$$

**Step2.** Define the second error variable:

(12) $$e_{2}=x_{2}-\rho$$

Taking the derivative with respect to the second error variable, we obtain:

(13) $${\dot{e}}_{2}={\dot{x}}_{2}-\dot{\rho }$$

Coupling Equations (9) with (11), we can obtain:

(14) $$\begin{array}{l} V_{1}=e_{1}{\dot{e}}_{1}\\ =e_{1}(x_{2}-{\dot{x}}_{d})\\ =e_{1}(e_{2}+\rho -{\dot{x}}_{d})\\ =-\varphi_{1}e_{1}^{2}+e_{1}e_{2} \end{array}$$

Define the second Lyapunov function:

(15) $$V_{2}=V_{1}+\frac{s^{2}}{2}$$

In Equation (15), the sliding mode surface is denoted as s. The conventional design often takes $s=e_{2}$ as the sliding mode surface. However, although the control algorithm of this linear sliding mode surface design can affect the convergence speed to a certain extent by adjusting the parameter when guiding the system state to approach and move along the sliding mode surface, this design cannot guarantee that the steady-state error of the system will converge to zero in a finite time, that is, the final convergence time of the steady-state error cannot be guaranteed exactly. Therefore, the linear sliding mode surface has some limitations in the convergence speed and accuracy, which needs further optimization and improvement. In order to improve the convergence speed and tracking performance of Equation (15), a novel non-singular terminal integral sliding mode strategy is introduced in this section. This sliding mode surface design not only effectively circumvents the singularity problem that may arise in conventional terminal sliding mode control, but also significantly enhances the tracking performance.

(16) $$s=\delta +\sigma {\left|\delta \right|}^{g}\mathrm{sgn}(\delta )+\beta {\left|e_{2}\right|}^{h}\mathrm{sgn}\left|e_{2}\right|$$

where $\delta =\int_{0}^{t}e_{2}dt$, σ, $\beta \in R^{+}$ are the parameters of the sliding mode surface;1<h<2; g>h. From Equation (16), it can be seen that when the systematic error variable is far away from the equilibrium point, the higher-order term of δ plays a major role; on the contrary, when the error variable is close to the equilibrium point, the higher-order term of $e_{2}$ plays a major role. The combination of the two can make the systematic error variable converge quickly to the equilibrium point along the sliding mode surface (s=0) in a finite time. Function sgn(δ) is expressed as Equation (17):

(17) $$\mathrm{sgn}(\delta )=\left\{\begin{array}{l} -1,\;\delta <0\\ 0,\;\delta =0\\ 1,\;\delta >0 \end{array}\right.$$

Associating Equations (4), (13) and (16), the derivative of the sliding mode surface s can be obtained as:

(18) $$\dot{s}=(1+\sigma g{\left|\delta \right|}^{g-1})e_{2}+\beta h{\left|e_{2}\right|}^{h-1}(D(x_{1},x_{2},\omega (t),\Delta_{r})+bu-\dot{\rho })$$

In order to increase the speed of convergence of the error variables of the USV heading control system (4), the following double power convergence law is employed:

(19) $$\dot{s}=(-l{\left|s\right|}^{p}\mathrm{sgn}(s)-\mu {\left|s\right|}^{q}\mathrm{sgn}(s)){\left|e_{2}\right|}^{h-1}$$

where $l,\mu \in R^{+}$ is the double power convergence law parameter; p>1, 0<q<1; $-l{\left|s\right|}^{p}\mathrm{sgn}(s)$ term and $-\mu {\left|s\right|}^{q}\mathrm{sgn}(s)$ term guarantee the dynamic quality of the systematic error variable away from the sliding mode surface $\left(\left|s\right|>1\right)$ and close to the sliding mode surface $\left(0<\left|s\right|\leq 1\right)$, respectively, and the combination of the two allows the systematic error variable to rapidly converge from the initial position s(0) to the sliding mode surface in a finite time (s=0).

By associating Equations (18) and (19), at the same time, in order to minimize the appearance of fractions in the formula, $\frac{e_{2}}{{\left|e_{2}\right|}^{h-1}}={\left|e_{2}\right|}^{2-h}\mathrm{sgn}(e_{2})$ is processed in the calculation, so the following non-singular terminal integral sliding mode backstepping controller for USV can be derived:

(20) $$\begin{array}{l} \tau_{r}=\frac{1}{b}[-\frac{1}{\beta h}[(1+\sigma g{\left|\delta \right|}^{g-1}){\left|e_{2}\right|}^{2-h}\mathrm{sgn}(e_{2})+\\ \left(l{\left|s\right|}^{p}+\mu {\left|s\right|}^{q}\mathrm{sgn}(s)])-D(x_{1},x_{2},\omega (t),\Delta_{r})+\dot{\rho }\right] \end{array}$$

From Equations (10) and (20), it can be seen that $\tau_{r}$ contains the derivative form of the virtual control quantity ρ, if the direct derivation may lead to the phenomenon of differential explosion, but also increase the complexity of the calculation, so this paper introduces the following second-order sliding-mode filter for its estimation.

(21) $$\left\{\begin{array}{l} {\dot{\upsilon }}_{1}=-\frac{\theta_{1}-\rho }{H_{1}}-\frac{\varsigma_{1}(\theta_{1}-\rho )}{\left|\theta_{1}-\rho \right|+E_{1}}\\ {\dot{\upsilon }}_{2}=-\frac{\theta_{2}-{\dot{\upsilon }}_{1}}{H_{2}}-\frac{\varsigma_{2}(\theta_{2}-{\dot{\upsilon }}_{1})}{\left|\upsilon_{2}-{\dot{\upsilon }}_{1}\right|+E_{2}} \end{array}\right.$$

where $H_{1}$, $H_{2}$$E_{1}$, $E_{2}$, $\varsigma_{1}$, $\varsigma_{2}$ are the filter constants to be designed and satisfy all positive real numbers, $\upsilon_{1}$ is the filtered value of ρ, and $\upsilon_{2}$ is the estimated value of $\dot{\rho }$.

Combining Equations (5) and (21), thus Equation (20) can be rewritten as

(22) $$\begin{array}{c} \tau_{r}=\frac{1}{b}\{-\frac{1}{\beta h}[(1+\sigma g{\left|\delta \right|}^{g-1}){\left|e_{2}\right|}^{2-h}\mathrm{sgn}(e_{2})+\\ \left(l{\left|s\right|}^{p}+\mu {\left|s\right|}^{q}\mathrm{sgn}(s)])-{\hat{x}}_{3}+\upsilon_{2}\right\} \end{array}$$

### 3.3. Stability and Convergence Analysis

**Lemma** **4.** *For the USV heading control system (4), by choosing a non-singular terminal integral sliding mode surface (16) and adopting the law of double power convergence (19), the error variable of system (4) can converge rapidly from the initial position to the equilibrium point in a finite time.*

**Proof.**  Assume that the convergence time of the arrival phase of the sliding mode surface in this paper is $t_{r}$ and the initial position of the error variable of the USV heading control system satisfies s(0)>1, then in the process of convergence of the system error variable from the initial position s(0) to s=1, the effect of the 1st term in Equation (19) $-l{\left|s\right|}^{p}\mathrm{sgn}(s){\left|e_{2}\right|}^{h-1}$ is much larger than the effect of the 2nd term-entry $-\mu {\left|s\right|}^{q}\mathrm{sgn}(s){\left|e_{2}\right|}^{h-1}$, so ignoring the effect of the 2nd term and taking it to be of the form $e_{2}(t_{r1})$, Equation (19) becomes

(23) $$\dot{s}=-ls^{p}{\left|e_{2}(t_{r1})\right|}^{h-1}$$

When the systematic error variable reaches the sliding mode surface s=0 from s=1, the second term in Equation (19) plays the main role, so the effect of the first term is neglected, and at the same time, $e_{2}$ is taken as the form of $e_{2}(t_{r2})$, then Equation (19) becomes

(24) $$\dot{s}=-\mu s^{q}{\left|e_{2}(t_{r2})\right|}^{h-1}$$

Integrating Equations (23) and (24), respectively, and considering that Equations (23) and (24) are obtained by neglecting one of the terms in Equation (19), the convergence time $t_{r}$ of the error variable of the USV heading system in the arrival phase satisfies:

(25) $$t_{r}<t_{r1}+t_{r2}=\frac{s{\left(0\right)}^{1-p}-1}{(1-p)l{\left|e_{2}(t_{r1})\right|}^{h-1}}+\frac{1}{(1-q)\mu {\left|e_{2}(t_{r})\right|}^{h-1}}$$

End proof. □

## 4. Simulation and Results Analysis

In this section, the proposed controller is simulated and compared with the BSMC (reference Remark 2). Reference [25] is selected as our simulation object. The parameters of USV are referenced in Table 1, and some key parameters of both controllers are listed in Table 2.

The simulation research is divided into two parts; one is heading control, one is path tracking control. In heading control simulation, we designed two different sets of experiments. Case 1 is designed to verify the tracking performance of the proposed method under the same random interference, respectively, under the tasks of fixed heading and changing heading, while Case 2 is designed to verify the tracking effect under the superposition of external interference. In path tracking, we carried out linear and curve tracking simulations, respectively. The specific simulation content is in Section 4.1 and Section 4.2.

**Remark** **2.** *In the design of traditional BSMC (backstepping sliding mode control), the sliding mode surface* $s=e_{2}$ *is selected, and the reaching law is selected as* $\dot{s}=-D_{1}\mathrm{sgn}(s)-h_{1}s,D_{1}>0,h_{1}>0$*, then the traditional backstepping sliding mode control law can be written as* $\tau_{BMC}=\frac{1}{b}(-P-D_{1}\mathrm{sgn}(s)-h_{1}s-c_{1}e_{1}+{\ddot{x}}_{d}-c_{2}e_{2}-e_{1})$*. In the formula,* $D_{1}$*,* $h_{1}$*,* $c_{1}$ *and* $c_{2}$ *are the controller parameters, and all are normal numbers;* $P=\frac{m_{1}-m_{3}}{m_{3}}uv-\frac{d_{3}}{m_{3}}r$*;* $e_{1}$ *is the heading Angle error, and* $e_{2}$ *is the virtual control quantity error.*

### 4.1. Heading Control Simulation Experiment

#### 4.1.1. Case 1

**Figure 3:**
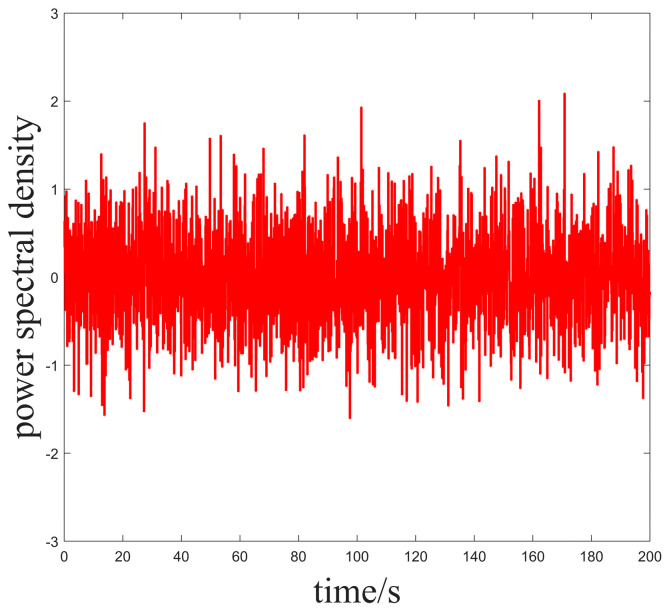
Power density spectrum of the noise added in Case 1.

In Case 1, we set the ideal heading angle of the USV to 30° and the initial heading angle of the actual USV to 0°; the external perturbation is set to be a random white noise disturbance to simulate the marine environment, as shown in Figure 3.

**Figure 4:**
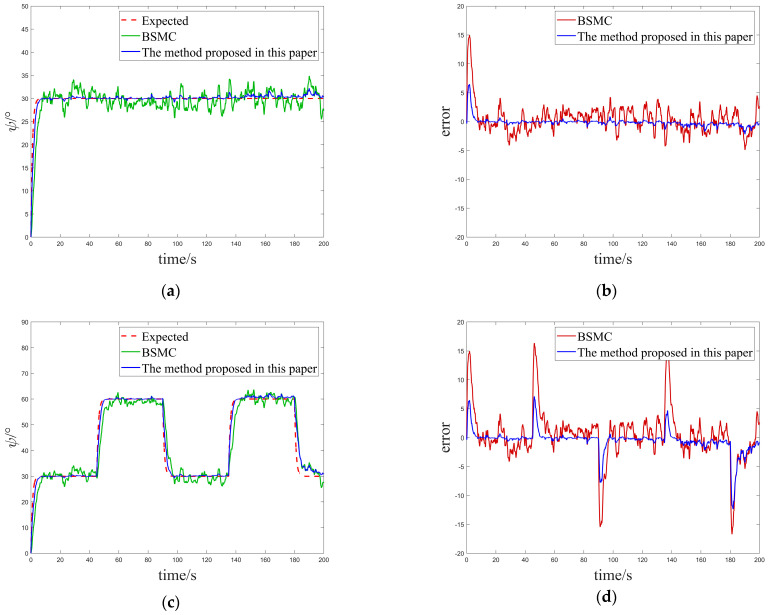
Comparison of the tracking effect and error for Case 1. (**a**) Comparison of control effects under constant heading. (**b**) Comparison of tracking errors under constant heading. (**c**) Comparison of control effects under square heading. (**d**) Comparison of tracking errors under square headings.

In this case, we focused on evaluating the performance of the control system under a single white noise disturbance. By carefully comparing the tracking results of the USV system in Figure 4a,c, it can be clearly observed that, both under constant heading commands and square sailing commands, the USV system exhibits faster and more accurate heading tracking capabilities as well as better interference immunity when using the innovatively designed algorithms in this paper compared to the conventional BSMC algorithms, which fully reflects its excellent dynamic response characteristics and high adaptability. To better visualize this advantage, we also comparatively analyze the tracking errors of the two algorithms under different desired headings, as shown in Figure 4b,d. These graphs visually show that the new controller proposed in this paper achieves a significant reduction in tracking error under white noise interference compared to the traditional BSMC algorithm, thus verifying its superior stability and robustness under complex environments and uncertainty conditions.

**Figure 5:**
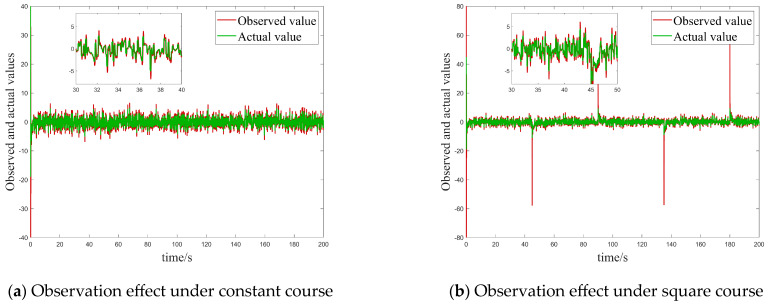
Observation effect of ESO on total disturbance for Case 1.

At the same time, it can be seen from Figure 5a,b that the designed ESO can easily observe lumped interference terms in the USV model.

**Remark** **3.** *When the command heading is given, we add a first-order low-pass filter to smooth the command signal, avoiding overshooting and the instability of the system due to direct response to rapidly changing commands, and ensuring a more supple and reliable control process.*

#### 4.1.2. Case 2

**Figure 6:**
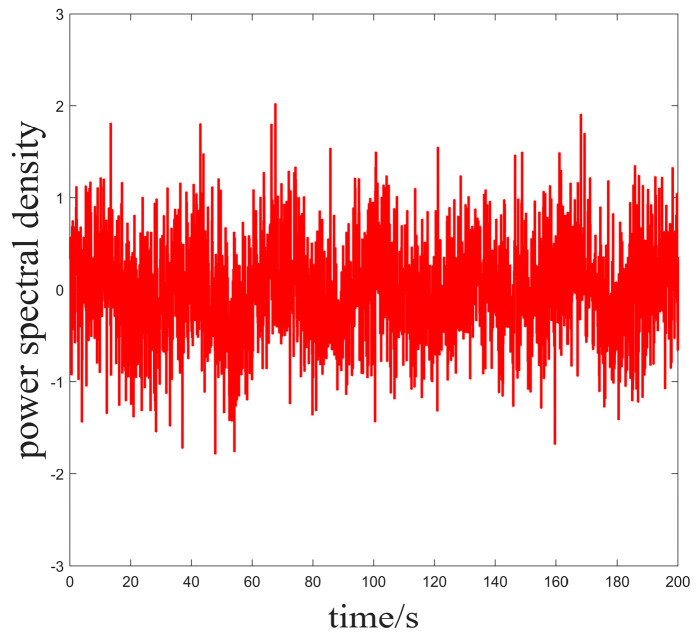
Interference noise added in Case 2 for Case 1.

In Case 2, in order to further verify the effectiveness of the proposed algorithm, we further upgraded the experimental conditions, to not only retain white noise interference, but also introduce slowly changing sine interference, so as to get closer to the multi-source compound interference environment that may be encountered in real navigation, as shown in Figure 6. The actual initial course angle of USV is 5 degrees; The ideal course angle setting for the USV is the same as in Case 1.

**Figure 7:**
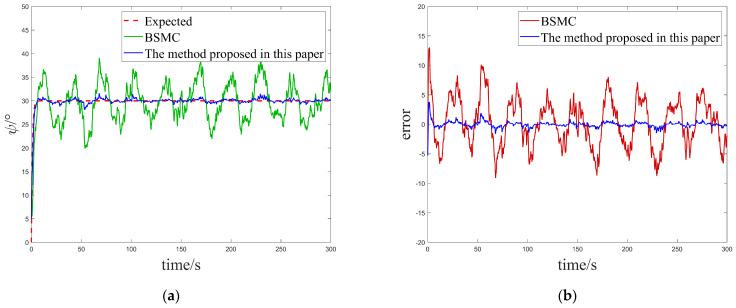

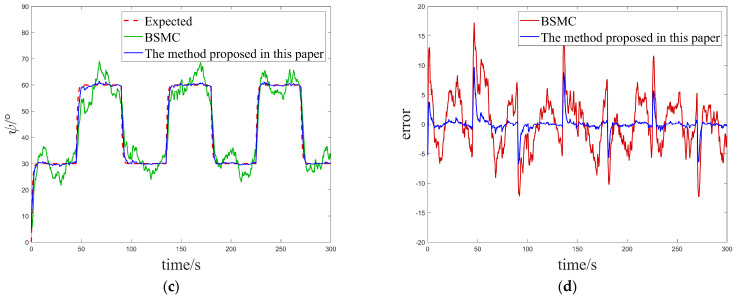
Comparison of tracking effect and error for Case 2. (**a**) Comparison of control effects under constant heading. (**b**) Comparison of tracking errors under constant heading. (**c**) Comparison of control effects under square heading. (**d**) Comparison of tracking errors under square headings.

**Figure 8:**
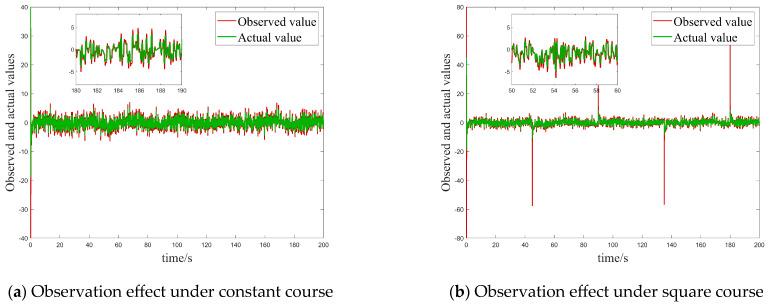
Observation effect of ESO on total disturbance for Case 2.

By observing Figure 7, we can see that in the second set of experiments, the experimental conditions are more stringent. However, through a comparative analysis of Figure 7a,c, we can find that the USV system can still quickly and accurately track the expected course in a short period of time by using the algorithm designed in this paper, which once again proves its good dynamic response speed and strong adaptive ability. In order to prove this result more intuitively, we also compare and analyze the tracking errors of the two algorithms under different expectation headings, (b) and (d). These comparison results further confirm that the new controller proposed in this paper can still maintain low tracking error in the face of more complex interference environments. The comparison between the two sets of experiments further strengthens the superior performance of the proposed new controller in the convergence rate and tracking error in the face of complex multi-source interference environment. And Figure 8a,b shows the lumped interference of ESO to the USV system under Case 2.

### 4.2. Path Tracking Simulation Experiment

**Figure 9:**
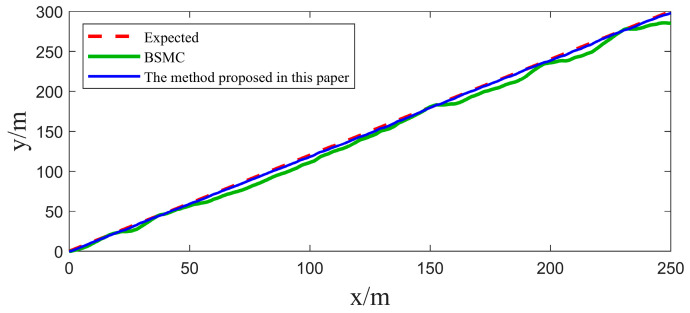
Comparison of USV linear path tracking results.

**Figure 10:**
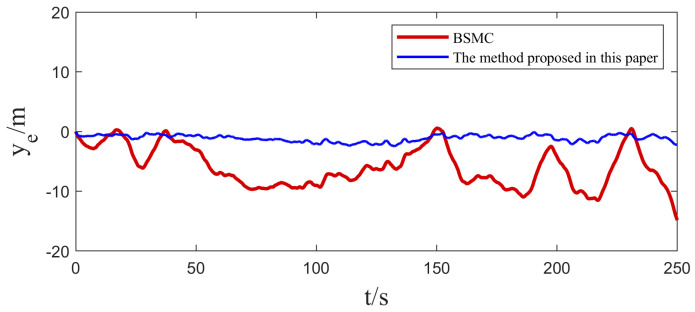
Comparison of USV linear path tracking errors.

**Figure 11:**
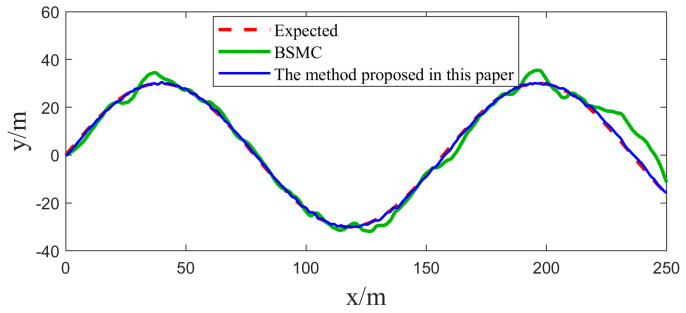
Comparison of USV curve path tracking effectiveness.

**Figure 12:**
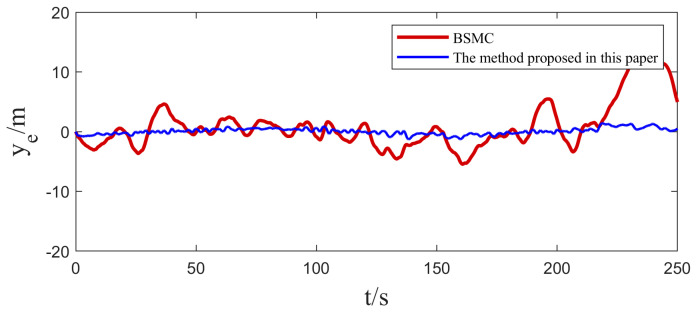
Comparison of USV curve path tracking errors.

In Section 4.1, we implemented two sets of comparative experiments on heading control for USVs. In order to further validate the practicality of the algorithms in this paper, two sets of path tracking experiments are further carried out in this section. The first group focuses on straight-line path tracking, while the second group focuses on curved path tracking, and the same Gaussian white noise is introduced as a disturbance factor in both sets of experiments. The experimental results and their corresponding tracking errors are shown in Figure 9, Figure 10, Figure 11 and Figure 12.

By carefully observing the path tracking effectiveness presented in Figure 9, Figure 10, Figure 11 and Figure 12, we can clearly conclude that both the traditional backstepping sliding mode control (BSMC) and the innovative algorithms proposed in this paper show satisfactory performances in meeting the basic requirements of system control performance. However, when comparing the control effects of the two in depth, it can be clearly seen that compared with the traditional BSMC algorithm, the algorithm proposed in this paper makes further significant improvements in the control performance of the system through the clever introduction of a new type of sliding mode surface and convergence law.

At the level of specific performance indicators (refer to Table 3 and Table 4), the numerical changes in the integral absolute error (IAE) and integral time absolute error (ITAE) strongly support the above conclusion. In the comparison experiments, the IAE and ITAE index values of the algorithm with the novel sliding mode surface and convergence law are significantly lower than those of the traditional backstepping sliding mode control alone, which directly proves that the algorithm proposed in this paper demonstrates a better performance in the reduction in the error accumulation and the accumulation of the error weighted with time.

From these simulation results, it can be seen that the ESO has a good real-time estimation of lumped disturbance, and the algorithm has the advantages of integrating terms that can more accurately reflect the dynamic characteristics of the system, and the convergence speed of the terminal sliding mode surface is faster. Therefore, the proposed algorithm system has better anti-interference ability and adaptive ability in USV tracking control. Therefore, the proposed algorithm has the advantages of easy implementation and good anti-interference effect, and it can be applied to the USV control system with uncertain nonlinearity and external disturbance.

## 5. Conclusions

Addressing the problem of accurate heading control and path tracking of USVs in complex dynamic environments, this study proposes an innovative control algorithm that integrates the extended state observer (ESO) with a non-singular terminal integral sliding mode control strategy. First, we design and implement the ESO, which is capable of estimating and compensating the total perturbations induced by the internal dynamics that are not modeled and the external environment of the USV system in real-time and accurately, substantially enhancing the control system’s adaptability to environmental changes. Additionally, we improve the traditional backstepping sliding mode controller by designing a novel non-singular terminal integral sliding mode surface. This innovative design not only possesses the fast convergence property of the terminal sliding mode (TSM), but also effectively avoids the singularity problem that may occur in the traditional TSM design. In addition, we introduce a second-order sliding mode filter (SOSMF) to accurately estimate the differential signals of the virtual control quantities, which reduces the computational complexity and avoids the differential explosion problem that may arise due to the successive differential operations in backstepping control, thus significantly enhancing the engineering practicability. The results of simulation experiments show that compared with the traditional backstepping sliding mode control method, the method proposed in this study not only significantly improves the convergence speed, but also achieves more accurate heading control and path tracking under complex and variable environmental disturbances. In summary, the control scheme proposed in this study achieves significant results in solving the USV heading control and path tracking problems and provides new ideas and methods for the field of intelligent control of USVs. Nevertheless, it is clear that the current research focus is on simulation experiments, and for further validation, future work should move to real-world testing with USV, while also taking into account more complex tasks and scenarios. In addition, including the possible saturation of actuators into the research category is also an important direction for future research.

## Figures and Tables

**Table 1:** USV three-degree-of-freedom model parameters.

Coefficient	Value	Coefficient	Value
$m_{1}$	2652.52	$d_{1}$	848.13
$m_{2}$	2825.57	$d_{2}$	10,162.44
$m_{3}$	4201.68	$d_{3}$	22,721.63

**Table 2:** The main parameters of the two controllers.

Algorithm	Parameters
BSMC	$c_{1}$$=1,\;c_{2}$ = 1 $D_{1}$$=0.6,\;h_{1}$ = 0.5
The algorithms in this paper	β=5.5, σ=6, p=2, q = 0.5 l=2.5, μ=5

**Table 3:** Linear path tracking error performance metrics statistics for USVs.

Evaluation Metrics	Algorithm	Value
IAE	BSMC	150.6
ITAE	The algorithms in this paper	67.2

**Table 4:** Performance metrics statistics for curved path tracking error in USV.

Evaluation Metrics	Algorithm	Value
IAE	BSMC	12,875.4
ITAE	The algorithms in this paper	10,224.9

## Data Availability

The data presented in this study are available on request from the corresponding author. The data are not publicly available due to privacy.
